# Cannabinoids in Medicine: Cancer, Immunity, and Microbial Diseases

**DOI:** 10.3390/ijms22010263

**Published:** 2020-12-29

**Authors:** Paweł Śledziński, Agnieszka Nowak-Terpiłowska, Joanna Zeyland

**Affiliations:** 1 Department of Genome Engineering, Institute of Bioorganic Chemistry, Polish Academy of Sciences, 60-032 Poznan, Poland; pawel717@gmail.com; 2 Department of Biochemistry and Biotechnology, Poznan University of Life Sciences, 60-632 Poznan, Poland; agnieszka.terpilowska@up.poznan.pl

**Keywords:** cannabinoids, cannabinoid receptors, cancer medicine, immune medicine

## Abstract

Recently, there has been a growing interest in the medical applications of *Cannabis* plants. They owe their unique properties to a group of secondary metabolites known as phytocannabinoids, which are specific for this genus. Phytocannabinoids, and cannabinoids generally, can interact with cannabinoid receptors being part of the endocannabinoid system present in animals. Over the years a growing body of scientific evidence has been gathered, suggesting that these compounds have therapeutic potential. In this article, we review the classification of cannabinoids, the molecular mechanisms of their interaction with animal cells as well as their potential application in the treatment of human diseases. Specifically, we focus on the research concerning the anticancer potential of cannabinoids in preclinical studies, their possible use in cancer treatment and palliative medicine, as well as their influence on the immune system. We also discuss their potential as therapeutic agents in infectious, autoimmune, and gastrointestinal inflammatory diseases. We postulate that the currently ongoing and future clinical trials should be accompanied by research focused on the cellular and molecular response to cannabinoids and *Cannabis* extracts, which will ultimately allow us to fully understand the mechanism, potency, and safety profile of cannabinoids as single agents and as complementary drugs.

## 1. Introduction

Over the last few years, there has been growing interest in the medical uses of plants of the genus *Cannabis* (hemp), both in the scientific community and among the general public. In many countries, efforts are made to loosen drug regulations, with a view on increasing access to cannabis-based medications. Additionally, much evidence from pre-clinical and clinical studies has been gathered over the last decade, suggesting that multiple substances produced by *Cannabis* plants have a therapeutic potential, including anticancer properties [1,2].

Advances in knowledge on *Cannabis* plant properties and their medicinal uses occurred despite an unfavorable legal landscape. Research on the secondary metabolites of *Cannabis* plants and medicinal uses of their derivatives has been—and in many places remains—severely restricted [3]. This is due to several reasons, the most important of which is the ban on growing *Cannabis* varieties other than the so-called industrial hemp—with a tetrahydrocannabinol (THC) content below 0.2% by dry weight, which entails no psychoactive properties and no addiction potential—as well as the multitude and variety of active substances produced by plants of this genus [4]. Recently, attention has largely shifted towards secondary metabolites of hemp other than cannabinoids. They may act synergistically with cannabinoids, providing beneficial therapeutic effects [5,6].

*Cannabis* L. is a genus of plants in the family *Cannabaceae* Endl. Its species produce unique secondary metabolites termed plant cannabinoids, or phytocannabinoids. Traditionally, three species of *Cannabis* were recognized: *C. sativa* L., *C. indica* Lam., and *C. ruderalis* Janisch. Recently, however, this taxonomic classification has been questioned, as it may not reflect the actual variability existing in this group of plants [7]. The popular belief about specific differences in psychoactive properties between *C. sativa* and *C. indica* is also false, as the biochemical composition of plants in both species is greatly variable [8]. Currently, researchers propose classifying all *Cannabis* plants as one species: *C. sativa*, with several chemotypes identified based on differences in the chemical composition of the plants [9].

Marijuana, also simply termed “cannabis”, is a drug derived from *Cannabis* plants. It is made from dried leaves and flowers of the psychoactive varieties of *C. sativa* and *C. indica*, which have a high concentration of psychoactive cannabinoids (mainly Δ9-THC) [10]. *Cannabis* plants are also used to produce hashish (pressed resin) and hemp oil. These products can be consumed by inhalation (smoking or vaporization) or ingestion (drinking or eating in a variety of preparations). Cannabinoid content in cannabis products may vary significantly based on the plant variety used, growing techniques, and type of product (oil, hashish, marijuana) [11]. Moreover, psychoactive cannabinoid content in *Cannabis* plants grown for drug production has increased over time owing to selection processes. A 2012 meta-analysis reported a significant and continuing increase of THC concentration in recreational cannabis since the 1970s [12]. Later studies have confirmed that the trend persists [13].

Cannabinoids are generally defined as lipophilic substances that act as ligands for a specific group of membrane receptors, termed cannabinoid (CB) receptors. They belong to the G-protein coupled receptor (GPCR) superfamily and constitute a part of the so-called endocannabinoid system. Cannabinoids are traditionally classified as plant cannabinoids (phytocannabinoids), endocannabinoids, and synthetic cannabinoids [14]. They are discussed in depth in the next section.

Medical potential of cannabinoids in a variety of conditions have been attracting increasingly more attention. An area which has been the most extensively explored include the use of cannabinoids in palliative care, mostly due to their analgesic and antiemetic properties related to their modulatory role in neurotransmission [15]. Their involvement in immunity and regulation of apoptotic and angiogenetic signaling pathways makes cannabinoids subject of research in the context of other ailments like cancer, inflammatory diseases, or microbial infections [11,16].

The aim of the review is to present the state of research exploring the cannabinoids’ therapeutic use as well as the current understanding of mechanism of action underlying these applications. We also identify obstacles on the pathway towards clinic, including gaps in knowledge, quality of studies and conflicting legal status.

## 2. Plant, Animal, and Synthetic Cannabinoids

Phytocannabinoids are a group of 21-carbon or 22-carbon (in carboxylated forms) terpene phenolic compounds produced by *Cannabis* plants as secondary metabolites [17]. To date, over 100 plant cannabinoids have been isolated and described, with the following found (in carboxylated forms) in the highest concentrations: tetrahydrocannabinolic acid (THCA), cannabidiolic acid (CBDA), cannabinolic acid (CBNA), cannabigerolic acid (CBGA), cannabichromenic acid (CBCA), and cannabinodiolic acid (CBNDA) [5]. THCA is the main cannabinoid in “psychoactive” *Cannabis* strains, while CBDA is dominant in industrial hemp [18].

The initial substrates in the biosynthesis of phytocannabinoids are geranyl diphosphate (geranyl pyrophosphate, GPP) and olivetolic acid (OLA). The enzyme geranyl-diphosphate:olivetolate geranyltransferase (GOT) catalyzes the alkylation of OLA by GPP, producing CBGA, the central precursor of multiple cannabinoids. In the next stage, the compound is transformed by oxide cyclases: tetrahydrocannabinolic acid synthase (THCAS) produces THCA, cannabidiolic acid synthase (CBDAS) produces CBDA, and cannabichromenic acid synthase (CBCAS) produces CBCA [5,17]. Over time the cannabinoid acids undergo non-enzymatic decarboxylation [5].

Among the known plant cannabinoids, Δ9-tetrahydrocannabinol is the primary psychoactive cannabinoid. Its effects on the human body consist in mimicking the endogenous cannabinoid receptor agonists, i.e., endocannabinoids [14]. THC intake produces a state of euphoria, as well as analgesic, antiemetic, and anti-inflammatory effects, though its psychoactive properties significantly restrict its medical uses [13]. Another phytocannabinoid produced in large quantities, which receives much attention from researchers, is cannabidiol (CBD). It displays a low affinity for CB receptors, meaning that its effects on the body are largely attributable to cannabinoid-receptor-independent mechanisms of action. CBD is known to interact with other receptors, including the transient receptor potential cation channel subfamily V member 1 (TRPV1), the orphan G protein-coupled receptors (GPR55, GPR119), and peroxisome proliferator-activated receptors (PPARs, especially PPARα, PPARγ) [14]. These receptors are phylogenetically unrelated to CB receptors but can respond to cannabinoids. The reclassification of some of these receptors as CB receptors has been proposed, though their exact role in endocannabinoid signaling remains unclear [14]. CBD has anxiolytic properties, and partially counteracts the psychoactive effects of THC [13].

Endocannabinoids are part of the so-called endocannabinoid system (ES) present in animals, which comprises CB receptors (CBRs), their endogenous ligands, and enzymes involved in their biosynthesis, transport, and degradation [19]. The most researched endocannabinoids are N-arachidonoylethanolamine (AEA, or anandamide) and 2-arachidonoylglycerol (2-AG). Both are derived from arachidonic acid and synthesized “on demand” in response to physiological stimuli, rather than stored in vesicles. Their levels are especially high in striatum and brainstem [20]. Other substances classified as endocannabinoids include virodhamine, N-arachidonoyl dopamine, and 2-arachidonoylglyceryl ether (noladin ether). Overproduction of endocannabinoids triggered by alcohol drinking may affect cardiovascular functions by CBR1 signaling [21]. Enzymes involved in endocannabinoid synthesis and degradation play an essential role in cellular signaling [22]. Nowadays, the endocannabinoid system, non-CB receptors involved in cannabinoids’ action, and endocannabinoid-like ligands are postulated to constitute a broader system, “endocannabinoidome” [10].

Synthetic cannabinoids (SCBs) are CB receptor ligands produced by chemical synthesis. They are mainly used in research on the relationship between cannabinoids’ structure and their activity, though they have also found some medicinal and recreational uses [23]. Their potential toxicity and the increase in recreational use has led, however, to public health concerns. Their health effects may be severe and include tachycardia, breathing disorders, and seizures [24]. Synthetic cannabinoids include JWH-018, HU-210, HU-331, SR144528, WIN 55,212-2, UR-144, and JWH-133, but more than 140 have been classified in this group. They are classified into four groups: aminoalkylindoles, classical cannabinoids, non-classical cannabinoids, and fatty acid amides [25]. SCBs can induce apoptotic cell death that is based on different mechanisms depending on the type of cannabinoid [24].

## 3. Cannabinoid Receptors (CBRs)

So far, two cannabinoid receptors (CBR1 and CBR2) with approx. 44% amino acid sequence homology have been identified in animal tissues [26]. Activation of each of these receptors entails adenylyl cyclase inhibition, resulting in a drop in cytoplasmic cyclic adenosine monophosphate (cAMP) concentration, Ca^2+^ channel closing, K^+^ channel opening, and the stimulation of protein kinases that play key roles in multiple signaling pathways, including the mitogen-activated protein kinase (MAPK), phosphoinositide 3-kinase (PI3K), or cyclooxygenase-2 (COX-2) pathways [27]. Expression of the CBR1 is the highest within the central nervous system (CNS), but it is broadly distributed and has also been identified in peripheral nerve endings and non-neural tissues, such as vascular endothelium, adipose tissue, lungs, liver, spleen, urinary bladder, prostate, testicles, and stomach [19]. In the brain, the CBR1 is particularly highly expressed in regions associated with the modulation of motor functions, memory, emotion, perception, and endocrine functions which explains the effects observed after THC administration or marijuana use [28].

One of the best understood roles of CBR1s in the CNS involves modulating neural stimulation through presynaptic inhibition. This involves a reverse signaling mechanism: endocannabinoids are synthesized and released by the postsynaptic neuron in response to neurotransmitter binding, and then diffused through the synaptic cleft to the presynaptic neuron membrane, where they bind to CB1 receptors, ultimately reducing the release of the neurotransmitter. As a result, presynaptic neuron stimulation leads to a considerably weaker response of the postsynaptic neuron [29]. Beside its neuromodulatory activity, the ES also plays a number of other important roles, e.g., in energy metabolism control, immune response, cardiovascular tension, and reproduction [30].

The CBR2 is mainly found on the surfaces of immune cell types, though its expression has also been observed in the CNS [31]. The potential of CBR2 in the therapeutic process of immunomodulation, the treatment of inflammatory and neuropathic pain, and neurodegenerative disorders is high [32]. CBR2 has a crucial role in immune balance and weakening the immune response.

Both endocannabinoids and exogenous cannabinoids have also been reported to interact with other types of receptors, especially GPR55 and GPR18 orphan receptors, and TRPV1, part of the transient receptor potential (TRP) family. The reclassification of some of these receptors as CB receptors, forming an integral part of the ES, has been proposed; however, this idea is countered by the fact that they also interact with endogenous and exogenous substances other than cannabinoids, and their exact role in functions of the ES remains unclear [33,34].

## 4. Cannabinoids in Medicine

**Figure 1:**
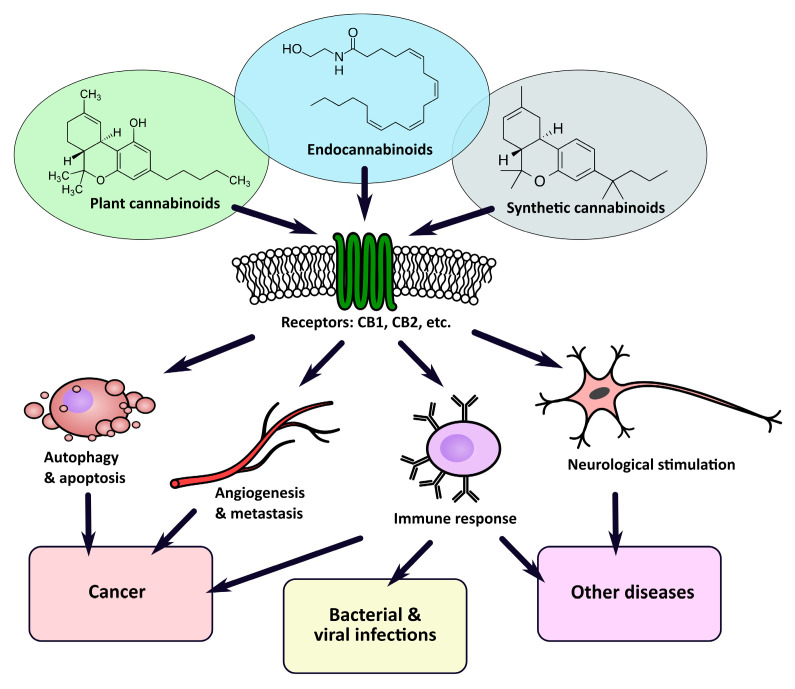
The involvement of the endocannabinoid system in a variety of modulatory processes makes it a promising target in the therapy of many conditions.

As the ES is involved in the regulation of multiple processes, including pathophysiological ones, it attracts a considerable amount of attention (Figure 1). Significant changes in ES activity have been observed in many pathological conditions, including cancers and neurological disorders such as Parkinson’s disease, Huntington’s chorea, and multiple sclerosis (MS). Therefore, the pharmaceutical modulation of ES, e.g., by administration of plant or synthetic cannabinoids, has become a promising therapeutic strategy in treating acquired immunodeficiency (AIDS)-associated weight loss or spasticity in MS and supporting palliative care (e.g., standardized plant extract nabiximols/Sativex^®^ or synthetic nabilone/Cesamet^®^ and dronabinol/Marinol^®^). Some amount of evidence show that oral cannabinoids may have a role in controlling chemotherapy-induced nausea and vomiting (CINV) but more studies have to be done to confirm this effect [35].

Cannabis stimulate appetite via CBR1 activity. Dronabinol is approved for the treatment of anorexia and weight loss in adult patients with HIV (*Human immunodeficiency virus*) but not in the cancer-related anorexia and weight loss [36]. CBR2 stimulation reduces some effects of inflammatory processes in HIV-infected patients [37]. Synthetic canabinnoid—ajulemic acid/anabasum—is being evaluated in double-blinded phase II clinical trials for the treatment of cystic fibrosis, diffuse cutaneous systemic sclerosis, dermatomyositis, and systemic lupus erythematosus [38]. Pre-clinical in vitro studies and animal model experiments have demonstrated that cannabinoids exhibit potential anticancer properties.

Considering the wealth of compounds produced by *Cannabis* plants (cannabinoids, terpenes, polyphenols, steroids, flavonoids, etc.), it is very likely that their extracts’ influence on humans is more complex than a simple addition of effects. These effects may involve multiple modes of action with diverse underlying mechanisms, such as modulating bioavailability, affecting cellular transport mechanisms, activating precursors to obtain products with a specific action or inactivating active components, contributing to different parts of the same signaling pathway by multiple components, or inhibiting the binding of a ligand to its target receptor. Two mechanisms have been proposed for the impact of CBD on THC pharmacokinetics: increasing membrane fluidity, which facilitates the penetration of THC into muscular tissue, and cytochrome P450 inhibition, which delays THC degradation [39]. CBD partially counteracts the adverse effects of THC, such as disturbed cognition and memory. Formulations containing THC and CBD may be safer and more easily accessible to patients than single THC. The stronger antispasmodic effects of cannabis extract was observed in MS patients [40,41].

The synergistic interactions of cannabinoids with terpenes and flavonoids produced by *Cannabis* have also been proven. Terpenes are known to modulate THC pharmacokinetics by increasing blood–brain barrier (BBB) permeability and absorption of transdermally administered substances [9]. Ratios between terpenoids and phytocannabinoids may substantially improve potential medical therapies [42]. Secondary metabolites may affect THC affinity for the CB1 receptor and interact with neurotransmitter receptors, which suggests an impact on the psychoactive effects of cannabinoids. Flavonoids may also potentially affect THC pharmacokinetics via cytochrome P450 inhibition. Some results suggest that a phytocannabinoid-terpenoid effect is not based on the CB1/CB2 receptors level but involves different molecular mechanisms in the neuronal circuits [43].

Despite some promising study results, the understanding of the specific interactions between cannabinoids and other *Cannabis* plant metabolites remains poor [6]. One challenge that is yet to be solved is the description of relationships between specific hemp chemotypes (and specific THC/CBD ratios) and the body’s response (both qualitative and quantitative) with regard to various health conditions. Thus, pre-clinical trials are required to accurately describe the interactions between *Cannabis* metabolites from the biochemical and pharmacological perspective, while clinical trials are necessary to estimate the efficacy and safety of optimal combinations of the relevant compounds.

### 4.1. Cancer Medicine

Changes in ES expression and activity occurring during carcinogenesis do not form a consistent pattern and vary considerably between specific cancer types. Depending on the tissue context, the ES may be significantly involved in disease progression or prevention, forming an interesting therapeutic target. Multiple experiments in a variety of models have been performed demonstrating that cannabinoids are capable of inhibiting cancer cell proliferation, spread, and angiogenesis since 1975 when the first report on their anti-cancer properties was published [44,45]. Some other reports indicate that in certain circumstances, cannabinoids can exhibit a carcinogenic potential [46,47,48].

Increased expression of CBRs and endocannabinoid concentrations have been observed in multiple cancers (skin, prostate, and colon cancers, hepatocellular carcinoma, endometrial sarcoma, glioblastoma, meningioma, pituitary adenoma, Hodgkin lymphoma, mantle cell lymphoma), but this is not always correlated with the expression of CBRs in tissues of origin [19,27,49,50]. Moreover, the expression of CBRs and enzymes involved in endocannabinoid metabolism is often associated with the aggressiveness of the cancer. This suggests that increases in ES activity may play a significant role in carcinogenesis [51,52]. *CBR1*/*CBR2* gene knockouts have been shown to considerably reduce the incidence of UV-induced skin cancers in mice [53]. Additionally, the CBR2 is a major regulator of the effects of the HER2 (human epidermal growth factor receptor 2) oncogene, and its overexpression results in increased vulnerability to leukemia induced by viral infection [54,55]. CBR2 expression level is the cancer prognostic factor and its high expression is connected with poor prognosis for patients with HER2-positive breast cancer and head/neck squamous cell carcinoma. CBR1 overexpression is associated with a bad clinical outcome for patients with pancreatic, prostate, ovarian, and colorectal cancers [22].

#### 4.1.1. Autophagy and Apoptosis

**Figure 2:**
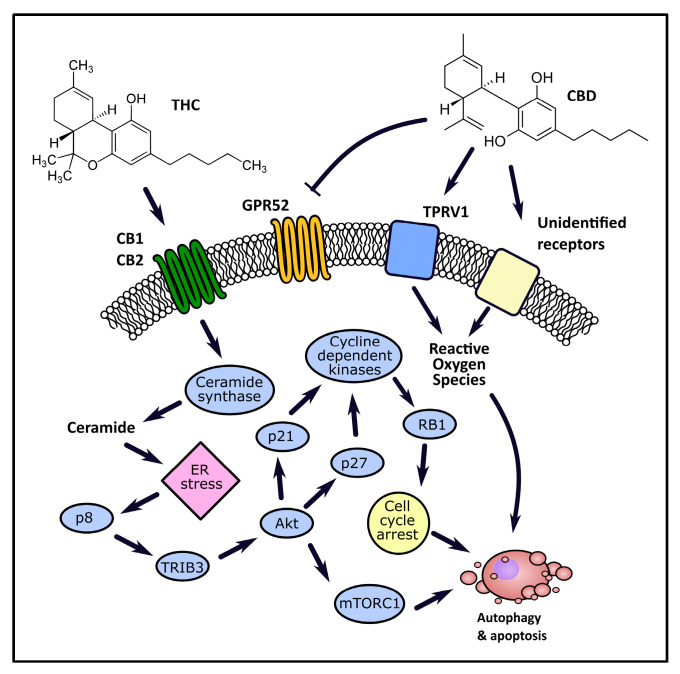
The overview of pathways involved in the anticancer activity of cannabinoids.

Endocannabinoids modulate extracellular signal-regulated kinase (ERK), p38 mitogen-activated protein kinase (MAPK), and the ceramide pathways in glioma, breast, prostate, and rectal cancer [56]. CBR agonists have been shown to stimulate glioma cell apoptosis by inducing de novo synthesis of compounds in the ceramide class—sphingolipids, which have proapoptotic properties [57,58]. The accumulation of ceramides activates signaling pathways associated with ER stress via CBR activation-dependent manner [59]. ER stress is associated with an increased expression of the stress-related nuclear protein 1 (Nupr1, or p8), a transcription regulator associated with carcinogenesis, and downstream elements of the signaling pathway: activating transcription factor 4 (ATF4), C/EBP homologous protein (CHOP), and tribbles pseudokinase 3 (TRIB3) protein [60,61]. The activation of the signaling pathway regulated by the p8 protein leads to protein kinase B (Akt) inhibition by TRIB3, which in turn inhibits the activity of mammalian target of rapamycin complex 1 (mTORC1), ultimately resulting in autophagic cell death. Figure 2 presents an overview of the cannabinoids’ anticancer action. Autophagy has been shown to precede apoptosis in the cascade of processes related to cell death that are triggered by cannabinoids. Autophagy inhibition prevents the induction of apoptosis by administered cannabinoids, while apoptosis inhibition prevents cell death, but not autophagy. Cannabinoids interacting with CBRs have been shown to induce autophagy in glioma, melanoma, liver, prostate, and pancreatic cancer cell lines [62,63,64,65].

Other mechanisms have also been proposed as contributors to the process of cannabinoid-induced cell death in some cell lines. In hepatocellular carcinoma, cannabinoid-induced ER stress may lead to AMP-activated protein kinase (AMPK) and calcium/calmodulin-dependent protein kinase kinase 2 (CAMKK2) activation, which is also considered a factor leading to autophagic cell death [64]. In experiments on breast cancer and melanoma cells, cannabinoid-induced Akt kinase inhibition leads to protein p21 and p27 activation, which in turn inhibit cyclin-dependent kinases (CDK), resulting in retinoblastoma protein (RB1) phosphorylation, and ultimately arresting the cell cycle and inducing apoptosis [66,67]. Similar results were obtained in experiments using prostate cancer cells. Administration of the WIN 55,212-2 cannabinoid led to p27 protein activation, CDK 4 inhibition, and RB1 phosphorylation, similarly arresting the cell cycle and inducing apoptosis. In gliomas and prostate cancer, a decrease in Akt activity may inhibit the phosphorylation of the Bcl-2-associated death promoter (BAD) protein, a pro-apoptotic member of B-cell lymphoma 2 (Bcl-2) protein family, which additionally contributes to the triggering of apoptosis [68,69]. A commonly proposed explanations of mechanism of action of cannabinoids with a low affinity for CBRs (i.a. CBD) involves the stimulation of reactive oxygen species (ROS) production, as the accumulation of ROS may trigger processes ultimately leading to cell death cell death by autophagy (Figure 2) [70,71,72,73]. Most research to date suggests that the mechanism of action of CBD and other non-psychoactive cannabinoids is unrelated to direct CBR activation. There are some reports indicating a role of interactions with other receptor types, such as GPR55, TRPV1, or TRPM8 (transient receptor potential cation channel subfamily M member 8). For instance, the anti-cancer effects of CBD and cannabigerol (CBG) have been shown to be partially associated with their antagonistic activity towards TRPM8 receptors [74]. On the other hand, some reports suggest that in certain circumstances CBD may induce cancer cell apoptosis, at least in part, through direct or indirect interaction with the CB2 receptor [75].

#### 4.1.2. Angiogenesis and Metastasis

Beside the antiproliferative and proapoptotic activity, cannabinoids also have other properties, which might potentially be significant in terms of their anticancer effects. These compounds have been shown to inhibit angiogenesis by blocking the vascular endothelial growth factor (VEGF) signaling pathway. CBR agonists decrease the expression of VEGF and its receptors 1 and 2 (VEGFR1, VEGFR2) in glioma and skin and thyroid cancers [76,77]. Moreover, as already mentioned, cannabinoids can inhibit endothelial cell proliferation, which is also induced by cancers, thus additionally contributing to cancerous angiogenesis inhibition [78]. These observations are consistent with experimental findings indicating that the pharmaceutical blocking of ceramide biosynthesis counteracts cannabinoid-induced inhibition of VEGF secretion and the resulting VEGFR2 activation [79].

Cannabinoids have also been shown to inhibit spontaneous and induced metastasis in animal models and restrict cancer cell invasion in vitro (in lung, breast, and cervical cancers, and gliomas). These effects are partially associated with the modulation of extracellular proteases and their inhibitors. They are also related, in a way that has not yet been described, to the underlying processes of cancer cell response to cannabinoids interacting with CBRs, as pharmaceutical inhibition of ceramide biosynthesis and reduction of p8 protein expression counteracts the described effects [78]. Some data show that low expression of CBR1 in colorectal cancer positively affects the metastatic process, inhibiting apoptosis and deregulating the main signaling pathways. These observations contribute to the idea that drugs directed at regulating the endocannabinoid system through the induction of CB1 receptor can be helpful in order to develop new anti-cancer therapies or improve existing ones [80].

Cannabinoids with a low affinity for CBRs, such as CBD, also have similar effects [81]. These are, however, linked to a reduced expression of the inhibitor of differentiation 1 (Id1) protein, which inhibits basic helix-turn-helix transcription factors and is a key regulator of metastatic potential in breast cancer [75]. Another mechanism for cancer invasiveness reduction and metastasis inhibition by CBD has also been proposed, based on studies of lung cancer cell lines (A549, H359, H460). It involves Intercellular Adhesion Molecule 1 (ICAM-1) stimulation leading to increased expression of the tissue inhibitor of matrix metalloproteinases-1 (TIMP-1), which is a key anti-invasion factor [82]. THC, CBD, and R(+)-methanandamide have been shown to stimulate ICAM-1 expression in lung cancer cell lines A549, H460, and metastatic lung cancer cells from a patient. This increases cancer cells’ susceptibility to lymphokine-activated killer (LAK) cell adhesion, followed by lysis [83].

### 4.2. Immune Medicine

The EC system plays a crucial role in immune system homeostasis. However, the immunomodulatory effects of cannabinoids have not yet been fully understood. The highest levels of CBR2 expression were found in B cells, natural killer (NK) cells, monocytes, granulocytes, and T cells [84]. Phytocannabinoids with a high affinity for the CBR2 exhibit immunomodulatory properties both in terms of the cellular and of the humoral response. An association has been reported between CBR2 expression level, cell activation, and the presence of immune modulators [85]. Inhibition of CBR2 with JTE907 acting as a selective CBR2 inverse agonist with combined *CBR1*/*CBR2* gene silencing, showed that CBR2 (not CBR1) is responsible for phytocannabinoid-mediated immune suppression [86]. CBR2 suppresses T cell immune responses in T cell-mediated diseases and at the same time positively regulates T-independent immune responses. CBR2 selective agonists have been shown to attenuate several autoimmune diseases in mice [87]. CBR2 inhibits immune cell activation and pro-inflammatory mediator release and is a possible target in treating diseases connected to these phenomena, mainly inflammatory and autoimmune diseases: juvenile idiopathic arthritis, inflammatory bowel disease, celiac disease, obesity and neuroinflammatory diseases [88,89]. For example, CBR2 agonist—JWH-015—was able to reduce obesity-associated inflammation in mice [90].

Significantly higher expression of the CBR1 has only been detected in T cells. Its activation has been suggested as a contributor to cannabinoid-induced polarization of cytokine secretion [91]. As expression of the CBR1 is the highest within the CNS and in peripheral nerve endings it is responsible for local controlling the levels of interleukin 1 beta (IL-1β) and cyclooxygenase-2 (COX2) having an anti-inflammatory effect [92]. Cytokines can act as pro- or anti-inflammatory agents and cannabinoids are able to modulate their release. Production of pro-inflammatory cytokines can be reduced by cannabinoids (IL-6 by anandamide, IL-12 and IFN-γ (interferon γ) by THC and CBR1, CBR2 partial agonists) [89]. Pro-inflammatory IL-2 and IFN-γ promote the differentiation of T-helper (Th) cells towards the Th1 subtype, while IL-4 and IL-5 promote differentiation towards the Th2 subtype. Cannabinoids are potent IL-10 inducers, and this anti-inflammatory cytokine has been associated with the suppression of Th1 response. This response is considered a key factor in effective immune response towards many types of cancer cells [93]. The effects of THC include inhibition of IFN-γ secretion, alteration of the Th1/Th2 profile, and suppression of T cell proliferation. CBD reduces inflammation through its antagonistic effects on CBRs, inhibiting immune cell migration [94]. CBD showed anti-inflammatory effects in a murine model of acute LPS-induced (lipopolysaccharide) lung injury associated with an increase in the extracellular adenosine offer and signaling through adenosine A(2A) receptor [95]. Inhibition of endocannabinoid degrading enzymes MAGL and FAAH results in lower number of adherent leucocytes [96]. Immunosuppressive effects of cannabinoids raise concerns about their attempted uses in cancer treatment. THC has been shown to inhibit the host’s immune response to cancer cells in a murine lung cancer model, consequently stimulating tumor growth, and similar findings have been reported in a murine breast cancer study [46].

#### Immune Thrombocytopenia (ITP)

ITP is an idiopathic or connected to other pathologies (i.a. drug-induced) autoimmune disease. There is a long list of drugs that can cause D-ITP (drug-induced thrombocytopenia): abciximab, carbamazepine, ceftriaxone, eptifibatide, heparin, ibuprofen, mirtazapine, oxaliplatin, penicillin, quinine, quinidine, rifampicin, suramin, tirofiban, trimethoprim–sulfamethoxazole, and vancomycin [97]. The main targets in ITP are circulating platelets that are being coated with autoantibodies and destroyed which leads to thrombocytopenia. At the same time, compensation mechanisms inducing platelets production by bone marrow are not efficient. ITP is characterized by abnormal cytokine secretion, especially by overproduction of pro-inflammatory IL-6 by mesenchymal stem cells. Natural activation of CBR2 or JWH-133 selective agonist implementation reduces IL-6 levels in ITP [98]. Substitution of glutamine (Q) with arginine (R) at the codon 63 in CBR2 protein changes its polarization and affects receptor–cannabinoid interactions reducing immune modulation function of this receptor [99]. There is a strong correlation between Q63R polymorphism and autoimmune disorders, especially ITP susceptibility in children [100].

### 4.3. Bacterial, Viral, and Parasite Infections

When it comes to pathogenic bacterial infections, it has been shown that exogenous cannabinoids, especially THC, can reduce resistance to bacterial infections like *Listeria monocytogenes*, *Treponema pallidum*, *Legionella pneumophila*, *Staphylococci aureus,* and *S. albus* in animal models suggesting the same outcome in humans [101]. T helper cell type 1 (Th1)-polarizing cytokines are induced by *Legionella pneumophila* infection and are suppressed by pre-treatment with THC in murine model. THC-induced suppression of Th1 polarization in response to *Legionella pneumophila* infection is not mediated by increases in corticosterone and prostaglandin E2 [102]. Periodontitis, a chronic bacterial-induced disease of the supporting structures of the teeth can be promoted by marijuana derived canabinnoids (CBD, CBN, and THC). Each of them is able to reduce IL-12 p40, IL-6, IL-8, and TNF release induced by oral pathogens (*Porphyromonas gingivalis, Filifactor alocis,* or *Treponema denticola)* while enhancing the anti-inflammatory cytokine IL-10 [89]. On the other hand, efficient inhibition of LPS, TNF-α, IL-1β stimulated IL-6, and MCP-1 production by CB2R ligands in hPDLFs (human periodontal ligament fibroblasts) can be promising for periodontal therapy, novel drugs development in context of improving oral health [103]. Marijuana consumption can increase severity of tuberculosis (TB) in *Mycobacterium tuberculosis* infections driven by THC impairment of innate immune response as well as it may worsen the condition of HIV-infected patients infected by *Acanthamoeba* (101). On the other hand, anti-inflammatory effects of cannabinoids can be used to reduce bacterial LPS-induced fever [104]. Plant cannabinoid (E)-BCP ((E)-β-caryophyllene), selective CBR2 agonist inhibits LPS-induced proinflammatory cytokine expression in peripheral blood and attenuates LPS-stimulated Erk1/2 and JNK1/2 phosphorylation in monocytes [99].

#### 4.3.1. Viral Infections

The development of a viral infection is the result of competition between the innate and adaptive immune system response of the organism and the infectious potential of the virus. The agonists of CBR1 can inhibit Ca^2+^ ions release, changing signal transduction and affecting an activation of Ca^2+^-dependent proteins [105,106]. There are numerous Ca^2+^-dependent enzymes like matrix metalloproteinases, transglutaminases, or calpains that play a role in complex inflammation processes. Malfunction of enzymes involved in the host’s immune protection can promote virus replication [101]. In various viral infections studied in vivo as well as in vitro, cannabinoids were able to increase the replication of the virus [107]. THC decreased host resistance to HSV-1 and HSV-2 (*Herpes simplex virus*), KSHV (*Kaposi’s sarcoma herpes virus*), and influenza [101]. On the other hand, THC administration decreased early mortality of male macaques infected by SIV (simian immunodeficiency virus), but the same effect was not observed for females. Progression of SIV was assessed among other changes in body weight, mortality, and viral levels [108]. Agonists of CBR2 have been shown to reduce infection in primary CD4+ T cells of HIV-1 (*Human immunodeficiency virus type 1*) that belongs to tropic viruses using CXCR4 (chemokine receptor type 4). CBR2 agonist decreases CXCR4-activation-mediated G-protein activity and MAPK phosphorylation. Selective agonist of CBR2—JWH133—decreased HIV replication by reduction in RT (reverse transcriptase) activity [109].

#### 4.3.2. SARS-CoV-2

SARS-CoV-2 belongs to the *Coronavirinae* family and is a sense RNA virus with envelope- and spike-like projections on its surface [110]. COVID-19 is often characterized by elevated inflammatory response manifested by C-reactive protein (CRP) overexpression, pro-inflammatory cytokines production (Il-6, IL-10, IL-1), higher TNF-α, neutrophil count, D-dimer, and blood urea [111]. The spike proteins of the virus bind to ACE2 (angiotensin-converting enzyme 2) receptors on the surface of the cell and release viral RNA into the cell via endocytosis. Host toll-like receptors (TLR3, 7, 8, and 9) bind to viral particles and detect hazard. Replicating viral particles stimulate immune system response leading to inflammation. Under some circumstances, immune response to SARS-CoV-2 can get out of body system control. Pro-inflammatory cytokines release in COVID-19 is called Cytokine Storm (CS). Macrophages are recruited by CoV-targeted cells (ACE2-expressing cells in lung, liver, stomach) during inflammation, and they play a defensive or destructive role in infection [112]. Macrophages and other inflammatory cells present in lungs cause CS leading to acute respiratory distress syndrome and death [113]. Moreover, SARS-CoV-2 predisposes or even causes bacterial co-infection leading to sepsis and worsening the clinical condition of the patient. As there is no COVID-19 unified treatment regimen yet, and most therapies are experimental and adopt drugs used to treat other syndromes, such as corticosteroids, remdesivir, or favipiravir, their effectiveness varies and is less predictable [114,115,116]. As mentioned before, CBR1 and CBR2 are important modulators of the immune system; therefore, we can assume that changes in the endocannabinoid system may play a role in the course of COVID-19 illness. ES modulation could be alternative for adopted “classical” anti-inflammatory drugs: non-steroidal and glucocorticoids, cytokines antagonists, monoclonal antibodies, or Janus kinase inhibitors [93]. It has been shown that women are less susceptible to SARS-CoV-2 infection because of estrogen production and their influence on CBR2 activity [117,118]. The link between ES and its regulation by estrogens is well known as they share the same molecular pathways. The modulation of ES, and in particular stimulation of CBR2, could be helpful in reduction of cytokines and antibodies production, giving these therapies against COVID-19 advantages over others, having extremely selective actions (like monoclonal antibodies).

#### 4.3.3. Parasite Infections

*Plasmodium falciparum* is a protozoan parasite responsible for about 50% of all malaria cases in humans transmitted through *Anopheles* mosquitoes. In 2018, there were 228 million cases of malaria worldwide reported and 405,000 deaths (World Health Organisation). Malaria parasites preferentially invade the erythrocytes of the host where they proliferate. The search for antimalarial drugs has led some researchers to check utility of cannabis in such therapies. 4-acetoxycannabichromene, 5-acetyl-4-hydroxycannabigerol, and -1′S hydroxycannabinol have showed mild-to-moderate antimalarial activity in vitro [119,120]. In mice infected with *Plasmodium* *berghei,* antiparasitic properties of *C. sativa* were not comparable with that of chloroquine, but results suggested the potential of cannabis in reducing pathogenicity and enhancing disease tolerance [121].

### 4.4. Gastrointestinal Inflammations

Q63R genetic variant and celiac disease was shown in children. This suggests that CBR2 is responsible for immune homeostasis in this autoimmune disease correlated with genetic predisposition and gluten intake [122].

Crohn’s disease (CD) and ulcerative colitis (UC) are inflammatory bowel diseases (IBDs) of not well known etiology. One of the explanations of a structural damage of the intestinal mucosa is the immune response against bacterial antigens within genetically predisposed people [123]. Treatment of IBD, depending on severity of disease, is mainly based on anti-inflammatory drugs (aminosalicylic acid, corticosteroids), immunosuppressants (azathioprine, methotrexate), biological drugs (TNF-α antibodies, vedolizumab, ustekinumab), or combination therapy. Side effects of combined therapies encourage the search for new alternative drugs/supplements (pro-, prebiotics, vitamins, cannabis).

Essential role of ES in regulating intestinal inflammation is indisputable. CBR1/CBR2 agonists significantly reduce experimental colitis [124]. Increased levels of anandamide significantly attenuate colitis in wild-type mice, but not in CBR1 and CBR2-deficient mice [125]. Pharmacological or genetic silencing of the FAAH gene prevented the development of colitis in animal model [126]. Human ex vivo studies are inconclusive, and extrapolation of results obtained in animal models should be careful [127]. Clinical trials carried out in persons with IBD did not improve biochemical parameters of CD patients treated with CBD. Both, in patients with CD and UC treated with CBD, botanical extract of cannabis or inhalations (cigarettes, vapors) quality of life outcomes were improved (weight gain, better appetite, and sleep) [128,129].

CBRs are abundant on the surface of hepatocytes, cholangiocytes, Kupffer, and stellate cells and ES is involved in regulation of liver homeostasis. Cirrhosis is characterized by liver haemodynamic dysregulation. Portal hypertension and systemic vasodilation lead to ascites, variceal bleeding, liver-related cardiomyopathy, and increased risk of cardiovascular events [10]. CBR1 antagonists are suggested to have potential of improving cardiovascular activity in cirrhosis, as it was shown that CBR1 contribute to cardiac contractility alterations related to liver cardiomyopathy in cirrhosis rat model [130]. CBR2 activation, unlike CBR1, can be protective against liver fibrosis. In a rat model of cirrhosis, JWH-133 (selective CBR2 agonist) promoted the regeneration of liver by slowing down the fibrosis process [131]. Due to the dualistic nature of interactions within the ES, treatment of liver cirrhosis based on this system should be carefully considered.

## 5. Conclusions

If research on cannabinoids is to progress to the clinical level, their potential adverse effects and conditions minimizing the risk of such adverse effects must be identified. There are reports that, under certain conditions, cannabinoids may stimulate cancer growth through immunosuppression, as previously described. Other concerns are related to the concentrations of cannabinoids produced in the body, and the relationship between these concentrations and the resulting response. In vitro, cannabinoids have been shown to stimulate cell proliferation at nanomolar concentrations and inhibit it at micromolar concentrations (biphasic response); notably, the latter considerably exceed concentrations found in the blood of cannabis smokers [85]. Additionally, sub-micromolar concentrations of CB receptor agonists have been reported to stimulate the proliferation of some cancer cell lines (glioma—U373-MG, lung cancer—NCI-H292). This effect was dependent on ADAM7 (a disintegrin and metalloproteinase domain 7) metalloproteinase activity, epidermal growth factor receptor (EGFR) activation, and the resulting stimulation of extracellular signal-regulated kinases (ERK) and Akt. The THC concentration used in that study (100–300 nM) was like those found in the blood after cannabis smoking or oral THC administration [48]. Similar effects were also found in the LNCaP prostate cancer cell line. THC, HU-210, and JWH-015 stimulated proliferation, with peak activity at a concentration of 100 nM [132]. Establishing cannabinoid responsiveness profiles of diverse cancers is, therefore, essential. If accomplished, it may extend the range of anticancer agents or complementary medications, allowing selection and adjustment of the treatment according to the vulnerability of cancer and to its associated risks.

The idea of combining cannabinoids with traditional anticancer drugs, for example, chemotherapeutics, to produce a synergistic effect seems interesting. CBD and/or THC can boost cytotoxicity of vinblastine (by downregulation of P-glycoprotein), mitoxantrone (by inhibition of ABCG2), cytarabine, doxorubicin and vincristine (by decreasing p42/44 MAPK activity), bortezomib, carmustine, doxorubicin (by upregulation of TRPV2 channels), temozolomide (activation of TRPV2 channels and autophagy), and carfilzomib (by reducing the proteasome β5i subunit) [133]. Promising results of studies on the combined use of temozolomide and THC in animal glioma models have prompted clinical trials of temozolomide–Sativex combination therapy [134,135,136]. Similar findings were reported in a study on pancreatic adenocarcinoma, where gemcitabine combined with cannabinoids synergistically inhibited cancer growth [137]. One benefit of combining cannabinoids with other treatments is that this approach could potentially allow for inhibiting cancer progression on multiple levels while minimizing toxicity, which is a major challenge in single-agent high-dose cytotoxic therapy. Initial studies on combination cannabinoid–radiation therapy also produced promising results [138]. Apoptosis induction by CBD through ROS generation may potentially enhance the DNA damage induced in cancer cells by traditional radiotherapy. Such an approach would allow for reducing radiation doses to minimize its adverse effects, while maintaining or even increasing the therapeutic effects. There is also a potential of combining gold nanoparticle mediated photodynamic therapy with CBD in breast cancer treatment [139].

So far, cannabinoids and cannabinoid-based formulations have mainly been applied in palliative care, for their analgesic and antiemetic properties, alleviation of the adverse effects of chemotherapy, and reducing spasticity in MS. Data collected in clinical trials to date are not unequivocal and do not deliver high-quality evidence in favor of cannabinoids and their preparations as effective treatment. Doubts about the quality of studies on medical cannabinoid uses have been expressed by authors of meta-analyses. Whiting et al. concluded that there is moderate evidence suggesting that cannabinoids may be used to manage chronic pain and spasticity, and low-quality evidence supporting their use in chemotherapy-induced nausea and vomiting, AIDS-related weight loss prevention, sleep disorders, and Tourette’s syndrome [140]. According to Rodriguez-Almaraz et al., there is low- to moderate-quality evidence supporting cannabinoids as an adjuvant in the treatment of brain cancer and there is low- to moderate-quality evidence suggesting cannabinoids are associated with higher survival rates in glioma patients [141]. A 2019 meta-analysis of cannabinoids in the treatment of mental health conditions indicated only very low-quality evidence for the use of cannabinoids in the treatment of anxiety disorders in patients with other medical illnesses [142]. The authors emphasized also issues related to the conducted studies, mainly lack of high-quality double-blinded randomized clinical trials, small sample sizes, variance in drug formulations, and problems with standardization across the studies.

The best currently available evidence supports the use of cannabinoids in the treatment of spasticity in multiple sclerosis. A 2018 meta-analysis of Torres-Moreno et al. included 17 randomized controlled clinical trials and demonstrated modest but significant effects of cannabinoids and no serious adverse effects [143]. An evidence-based guideline of the American Academy of Neurology describes oral cannabis extracts, THC, and Sativex as effective for reducing patient-reported spasticity symptoms and pain [144].

These qualified conclusions mean that further research will be needed if cannabinoid-based formulations are to be introduced into clinical practice. Detailed safety and efficacy analyses are vital, especially considering the difficulties involved in estimating the risk and benefit of these substances in many cases. Aspects that undoubtedly still require proper investigation include optimal administration routes and dosage, interactions, and side effects of cannabinoids. Another significant issue is the lack of molecular biomarkers (e.g., gene polymorphisms associated with response to cannabinoids) that would allow predicting the effectiveness of cannabinoid therapy in a specific patient.

The objective assessment of the medical potential of cannabis and its preparations is still challenging due to a conflux of issues: social stigma, on the one hand, and misrepresentation of their potential by alternative medicine proponents, on the other hand [145], conflicting legal status (33 states have approved cannabis medical applications, whereas DEA still classifies cannabis as a Schedule I drug, with no medical use and high potential for abuse [146]), as well as the recognized health risk associated with THC, particularly in children and in adolescents.

The recent dramatic spread of SARS-CoV2 is driving the search for new diagnostic, treatment, and preventing approaches. Consequently, it led to the reconsideration of cannabinoids as direct or indirect antiviral agents. As discussed earlier, cannabinoid activity may play a role in the modulation of host response towards viral infection, but there are no studies that directly assess the efficacy of cannabis upon viral illnesses. Specifically, the significance of the anti-inflammatory activity of cannabinoids in the context of viral infection is ambiguous. In cases of excessive, pathogenic inflammatory response of the host organisms towards the virus, as in Covid-19, the cannabinoids’ action may be beneficial. On the other hand, it may lead to a decrease in the host immune response, promoting infection and disease progression.

The administration route has a significant impact on the pharmacokinetics, bioavailability, and thus, effectiveness of a substance. Cannabinoids have poor water-solubility, which greatly limits the possibility of intravenous administration. Oral administration entails the issue of cannabinoid degradation in the acidic environment of the stomach. In turn, inhalation raises justified concerns about its adverse impact on the respiratory system and difficulties in precise dosage. One possible solution to the problem would be administering cannabinoids directly into the tumor mass. This method was used in the pilot study by Guzman et al., where patients with recurrent glioblastoma were treated with intracranial THC [50]. The approach is promising, as it would allow for using high concentrations, while reducing systemic side effects, but it would be limited by the location of the specific tumor in the body. An unfavorable location could interfere with or even prevent the use of this type of therapy. Modifications of the above method have also been proposed, involving the use of nanoparticles or biomaterials designed to release cannabinoids under specific conditions, such as the pH level of the tumor’s microenvironment, or radiation of a specific wavelength [147,148].

The future and ongoing clinical trials should be accompanied by basic mechanistic studies aimed at cellular responses to cannabinoids, and cannabis extracts. This endeavor will allow ultimately to fully understand the mechanism of action and to reliably assess the potency and safety profile of cannabinoids as single agents and as complementary medications.

## Data Availability

Not applicable.

## Abbreviations

THC	tetrahydrocannabinol
CB	cannabinoid
THCA	tetrahydrocannabinolic acid
CBDA	cannabidiolic acid
CBNA	cannabinolic acid
CBGA	cannabigerolic acid
CBCA	cannabichromenic acid
CBNDA	cannabinodiolic acid
GPP	geranyl pyrophosphate
OLA	olivetolic acid
GOT	geranyl-diphosphate:olivetolate geranyltransferase
THCAS	tetrahydrocannabinolic acid synthase
CBDAS	cannabidiolic acid synthase
CBCAS	cannabichromenic acid synthase
TRPV1	transient receptor potential cation channel subfamily V member 1
GPR	G protein-coupled receptors
PPARs	peroxisome proliferator-activated receptors
ES	endocannabinoid system
CBRs	CB receptors
AEA	N-arachidonoylethanolamine
2-AG	2-arachidonoylglycerol
SCBs	Synthetic cannabinoids
GPCR	G-protein coupled receptor
cAMP	adenosine monophosphate
MAPK	mitogen-activated protein kinase
PI3K	phosphoinositide 3-kinase
COX-2	cyclooxygenase-2
CNS	central nervous system
TRP	transient receptor potential
MS	multiple sclerosis
CINV	chemotherapy-induced nausea and vomiting
BBB	blood–brain barrier
HER2	human epidermal growth factor receptor 2
FAAH	fatty acid amide hydrolase
MAGL	monoacylglycerol lipase
VEGF	vascular endothelial growth factor
ERK	extracellular signal-regulated kinase
MAPK	p38 mitogen-activated protein kinase
Nupr1	stress-related nuclear protein 1
ATF4	activating transcription factor 4
CHOP	C/EBP homologous protein
TRIB3	tribbles pseudokinase 3 protein
mTORC1	mammalian target of rapamycin complex 1
ER	endoplasmic reticulum
TRP	transient receptor potential channel
GPR	G protein-coupled receptor
AMPK	AMP-activated protein kinase
CAMKK2	calcium/calmodulin-dependent protein kinase kinase 2
CDK	cyclin-dependent kinases
BAD	Bcl-2-associated death promoter
Bcl-2	B-cell lymphoma 2 protein
ROS	reactive oxygen species
TRPM8	transient receptor potential cation channel subfamily M member 8
CBG	cannabigerol
Id1	inhibitor of differentiation 1 protein
ICAM-1	intercellular adhesion molecule 1
TIMP-1	tissue inhibitor of matrix metalloproteinases-1
LAK	lymphokine-activated killer
A549	lung cancer cell lines
EC	endocannabinoid
IL	interleukin
COX2	cyclooxygenase-2
IFN-γ	interferon γ
Th	T-helper
LPS	lipopolysaccharide
Th1	T helper cell type 1
hPDLFs	human periodontal ligament fibroblasts
TB	tuberculosis
HSV-2	Herpes simplex virus
KSHV	Kaposi’s sarcoma herpes virus
SIV	simian immunodeficiency virus
HIV-1	human immunodeficiency virus type 1
CXCR4	chemokine receptor type 4
RT	reverse transcriptase
CRP	C-reactive protein
ACE2	angiotensin-converting enzyme 2
TLR	toll-like receptors
SARS	severe acute respiratory syndrome
MERS	middle east respiratory syndrome
CS	cytokine storm
D-ITP	drug-induced thrombocytopenia
CD	Crohn’s disease
UC	ulcerative colitis
IBDs	inflammatory bowel diseases
U373-MG	glioma cel line
NCI-H292	lung cancer cel line
ADAM7	disintegrin and metalloproteinase domain 7
EGFR	epidermal growth factor receptor
ERK	extracellular signal-regulated kinases
LNCaP	prostate cancer cell line
